# Retraction: Abdallah MS, Mosalam EM, Hassan A, et al. Pentoxifylline as an adjunctive in treatment of negative symptoms in chronic schizophrenia: A double‐blind, randomized, placebo‐controlled trial. CNS Neurosci. Ther. 2023;29:354–364 (https://doi.org/10.1111/cns.14010)

**DOI:** 10.1111/cns.14668

**Published:** 2024-03-11

## Abstract

**Retraction:** Abdallah MS, Mosalam EM, Hassan A, et al. Pentoxifylline as an adjunctive in treatment of negative symptoms in chronic schizophrenia: A double‐blind, randomized, placebo‐controlled trial. *CNS Neurosci. Ther.* 2023;29:354–364 (https://doi.org/10.1111/cns.14010)

The above article, published online on November 07, 2022 in Wiley Online Library (wileyonlinelibrary.com) has been retracted by agreement between the journal's Editor‐in‐Chief, Xiaoming Hu, and John Wiley and Sons Ltd. Following publication, the Editor‐in‐Chief raised concerns about the integrity of the clinical trial. An investigation revealed significant issues regarding the reliability of the reported data. As a result, the Editor‐in‐Chief no longer has confidence in the validity of the results presented in this article.

**Retraction:** Abdallah MS, Mosalam EM, Hassan A, et al. Pentoxifylline as an adjunctive in treatment of negative symptoms in chronic schizophrenia: A double‐blind, randomized, placebo‐controlled trial. *CNS Neurosci. Ther.* 2023;29:354–364 (https://doi.org/10.1111/cns.14010)

The above article, published online on November 07, 2022 in Wiley Online Library (wileyonlinelibrary.com) has been retracted by agreement between the journal's Editor‐in‐Chief, Xiaoming Hu, and John Wiley and Sons Ltd. Following publication, the Editor‐in‐Chief raised concerns about the integrity of the clinical trial. An investigation revealed significant issues regarding the reliability of the reported data. As a result, the Editor‐in‐Chief no longer has confidence in the validity of the results presented in this article.

